# Perfusion imaging using rubidium-82 (^82^Rb) PET in rats with myocardial infarction: First small animal cardiac ^82^Rb-PET

**DOI:** 10.1007/s12350-016-0564-6

**Published:** 2016-06-14

**Authors:** Andreas Ettrup Clemmensen, Adam Ali Ghotbi, Rasmus Poul Bodholdt, Anne Mette Fisker Hag, Philip Hasbak, Rasmus Sejersten Ripa, Andreas Kjaer

**Affiliations:** Departments of Clinical Physiology, Nuclear Medicine & PET and Cluster for Molecular Imaging, Rigshospitalet and University of Copenhagen, Rigshospitalet, Blegdamsvej 9, 2100 Copenhagen, Denmark

## Introduction

Assessing myocardial perfusion using ^82^Rb-PET is emerging as a valuable clinical tool.^1^,^2^ The rapid decay (*T*_½_ = 76 s) allows for absolute quantification of both rest and stress perfusion within 30 minutes. In addition to evaluation of epicardial disease with perfusion defects, also evaluation of balanced coronary and small vessel disease is possible. For further evaluation of how ^82^Rb-PET can be used clinically, pre-clinical application of the method would be valuable. However, so far no data on the use of ^82^Rb-PET in small animals have been published nor has the use of ^82^Rb-PET, to the best of our knowledge, been successfully tried. Therefore, we wanted to develop and test the applicability of the method in rats, despite the high positron range of ^82^Rb. To do so, we adapted the clinical method and tested it in rats with experimentally induced myocardial infarction.

## Case Summary

**Figure 1:**
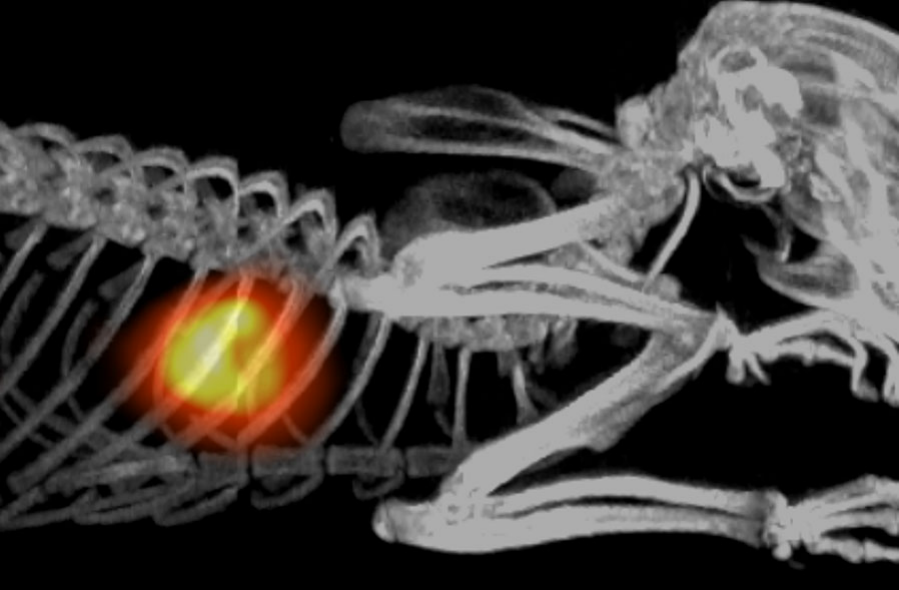
Fusion Maximum Intensity Projection (MIP) of ^82^Rb-PET/CT scan of a Male Sprague-Dawley rat after myocardial infarction LAD ligation, showing excellent cardiac uptake with practically no background uptake apart from clearance through the kidneys. The animal which was anesthetized using 4 % Sevoflurane in 35 % O_2,_ had a catheter placed in the tail vein, and was placed in a Siemens Inveon pre-clinical PET/CT scanner (Siemens, Knoxville, US). PET acquisition was performed for 5 minutes, with infusion of approximately 40 MBq ^82^Rb in 2 ml saline over 4-5 seconds from a clinically approved ^82^Rb generator (CardioGen-82, Bracco Diagnostics Inc., US) simultaneous with PET imaging, followed by a CT scan for anatomical co-registration and attenuation correction. PET list-mode data were histogrammed into two timeframes, the first 45 seconds and the remaining 255 seconds (the latter is shown here), and images were reconstructed using scatter and attenuation corrected OP-MAP / 3D-OSEM with 3 mm requested resolution, 18 and 2 subsets, respectively. All experiments were approved by the Danish Animal Experiments Inspectorate (Permit No. 2012-15-2934-00064)

A male Sprague-Dawley rat underwent ^82^Rb-PET/CT as described in Figure 1; after the baseline scan, the animal was subjected to myocardial infarction; a thoracotomy was performed, and LAD was ligated proximally. After closure, the animal recovered under anesthesia for 45 minutes, before being scanned again following the same protocol.

**Figure 2:**
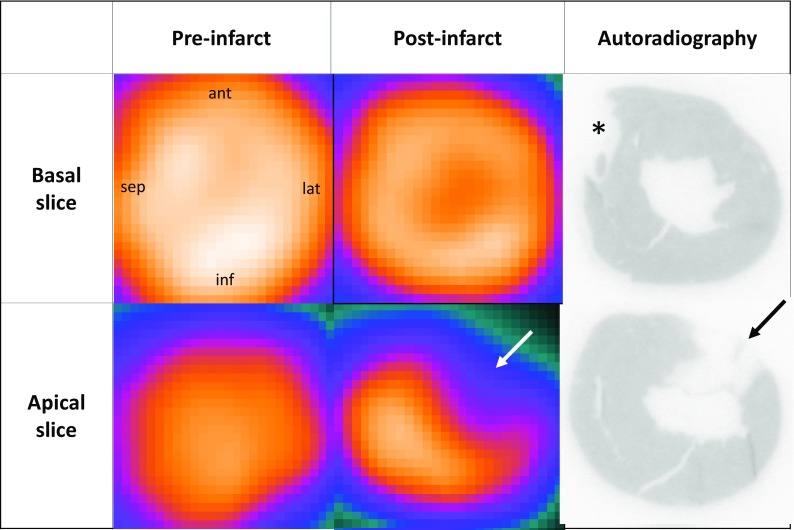
^82^Rb-PET short axis images of the heart before (left column) and after (middle column) LAD ligation. The reconstructed scan was imported into pMod software (v 3.3, pMod Technologies Ltd, Zürich, Switzerland) and reoriented to short axis view. After the second PET/CT scan, the animal was injected with 40-50 MBq of ^99m^Tc-Sestamibi and after 10 minutes the animal was euthanized by decapitation. The heart was rapidly excised and snap-frozen in liquid nitrogen. The frozen heart was casted in Tissue-Tek (Sakura, NL) and 20 slides (8 µm thick) were made, 240 µm apart, covering the infarcted area. The slides were exposed to a radiosensitive phosphor film and developed in a phosphor imager (Cyclone Plus, PerkinElmer Inc., US). The right panels show the resulting ex vivo autoradiography images of histological slices corresponding to the PET images. The infarcted area is marked by an arrow on both the ^82^Rb-PET and the autoradiography. The star (*) denotes a slicing artifact

Figure 2 shows the perfusion images, demonstrating clear uptake in the myocardium. On the post-infarction images, the perfusion defect is identified. The anterolateral location of the myocardial infarct was similar to that seen in humans when the culprit lesion is in the LAD. This despite anatomical differences between rats and humans.^3^ The myocardial infarct was subsequently confirmed by ex vivo autoradiography.

Despite the challenge of high energy of the positron emitted by ^82^Rb, the infarcted area could be identified on the in vivo images.

## Conclusion

For the first time, feasibility of ^82^Rb-PET in small animal cardiac imaging has been demonstrated. The method could delineate an infarcted area of a rat heart in vivo. These encouraging data stimulate for further development of the method.
